# Multidrug Resistant *Acinetobacter baumannii* Biofilms: Evaluation of Phenotypic–Genotypic Association and Susceptibility to Cinnamic and Gallic Acids

**DOI:** 10.3389/fmicb.2021.716627

**Published:** 2021-09-17

**Authors:** Mahmoud M. Sherif, Walid F. Elkhatib, Wafaa S. Khalaf, Nooran S. Elleboudy, Neveen A. Abdelaziz

**Affiliations:** ^1^Department of Microbiology and Immunology, Faculty of Pharmacy, Ahram Canadian University, 6th of October City, Egypt; ^2^Department of Microbiology and Immunology, Faculty of Pharmacy, Ain Shams University, Cairo, Egypt; ^3^Department of Microbiology and Immunology, Faculty of Pharmacy, Galala University, Al Galala, Egypt; ^4^Department of Microbiology and Immunology, Faculty of Pharmacy (Girls), Al-Azhar University, Cairo, Egypt

**Keywords:** *Acinetobacter baumannii*, biofilm, correlation, multidrug resistance, cinnamic acid, gallic acid

## Abstract

*Acinetobacter baumannii* armed with multidrug resistance (MDR) and biofilm-forming ability is increasingly recognized as an alarming pathogen. A deeper comprehension of the correlation between these two armories is required in circumventing its infections. This study examined the biofilm-forming ability of the isolates by crystal violet staining and the antibiotic susceptibility by broth microdilution method. The genetic basis of the MDR and biofilm-forming phenotypes was screened by polymerase chain reaction. The antimicrobial activities of cinnamic and gallic acids against planktonic cells and biofilms of *A. baumannii* were investigated, and the findings were confirmed with scanning electron microscopy (SEM). Among 90 *A. baumannii* isolates, 69 (76.6%) were MDR, and all were biofilm formers; they were classified into weak (12.2%), moderate (53.3%), and strong (34.5%) biofilm formers. Our results underlined a significant association between MDR and enhanced biofilm formation. Genotypically, the presence of *bla*_VIM_ and *bla*_OXA–23_ genes along with biofilm-related genes (*omp*A, *bap*, and *csu*E) was statistically associated with the biofilm-forming abilities. Impressively, both gallic and cinnamic acids could significantly reduce the MDR *A. baumannii* biofilms with variable degrees dependent on the phenotype–genotype characteristics of the tested isolates. The current findings may possess future therapeutic impact through augmenting antimicrobial arsenal against life-threatening infections with MDR *A. baumannii* biofilms.

## Introduction

*Acinetobacter baumannii* is receiving considerable attention as a troublesome pathogen owing to its extensive resistance to nearly all commonly used antimicrobials (Elkhatib et al., 2019), in addition to carbapenems, which are usually reserved to combat multidrug resistant (MDR) isolates (Xie et al., 2020). Carbapenem-resistant *A. baumannii* (CRAB) is ranked first priority by the World Health Organization (WHO) as a critical pathogen that urgently needs novel antimicrobial therapeutic strategies (WHO, 2017). Moreover, Centers for Disease Control and Prevention (CDC) has described CRAB as an “urgent threat” (CDC, 2019). During the coronavirus disease 2019 (COVID-19) pandemic, *A. baumannii* has added to the toll by causing fatal ventilator-associated pneumonia (VAP) outbreaks in COVID-specific intensive care units (ICUs) (Gottesman et al., 2021). Carbapenem-hydrolyzing *β*-lactamase genes, chromosomal or plasmid mediated, are the most common mechanism of carbapenem resistance in *A. baumannii* comprising *bla*_OXA–23,_ *bla*_OXA–24,_ *bla*_OXA–51,_ *bla*_OXA–58,_ *bla*_NDM,_ *bla*_VIM_, and *bla*_IMP_ (Lee et al., 2017). The high transmissibility of *A. baumannii* in hospital settings is greatly aided by its biofilm-forming capacity (Asaad et al., 2021). Biofilms offer protection to pathogens in the face of external stressors. Consequently, such infections respond inconsistently to antimicrobial treatments (Vestby et al., 2020). Genetic determinants related to biofilm include the biofilm-associated protein encoded by the *bap* gene, the outer membrane protein A (*omp*A), the pilus-like bundle structure mediated by the *csu*E gene, and *bla*_PER–1_ belonging to the *β*-lactamase family (Yang et al., 2019). Nonetheless, the interplay between biofilms and antimicrobial resistance of MDR *A. baumannii* isolates is rather disputable. Some researchers proved that *A. baumannii* expressed high levels of resistance to antibiotics despite producing weak biofilms (Qi et al., 2016), while others reported a positive correlation between antimicrobial resistance and biofilm-forming ability in MDR *A. baumannii* isolates (Bardbari et al., 2017).

In light of this combined problem, various approaches are implied to control spreading of biofilm-forming MDR *A. baumannii* strains (Farhadi et al., 2019). Lately, deployment of polyphenols is rising in popularity as a safe antibacterial and antibiofilm strategy (Zhang et al., 2019). Phenolic acids, such as gallic and cinnamic acid derivatives, cause irreversible changes in microbial membrane properties resulting in leakage of essential intracellular constituents (Kang et al., 2018). Concerning their antibiofilm activity, phenolic acids are being screened for “quorum quenching” abilities to disrupt quorum-sensing signals. Quorum sensing (QS) aids biofilm formation, rendering it a potential target for antibiofilm agents (Zhang et al., 2020).

There is a pressing need to comprehensively understand the nature of biofilms in *A. baumannii*, hence developing innovative and effective drugs to control such resistant infections. Accordingly, our study aimed to assess the relationship between biofilm formation and phenotypic–genotypic antibiotic resistance patterns in clinical isolates of *A. baumannii*. Besides, estimating the dissemination of biofilm-related genes with an attempt to pinpoint key ones serving as a predictor of strong biofilm formation phenotype. In addition, we investigated the antimicrobial and antibiofilm activities of cinnamic and gallic acids on MDR *A. baumannii* with a focus on underlying aspects influencing their effects.

## Materials and Methods

### Bacterial Isolates

In this study, previously identified and stocked clinical *A. baumannii* isolates (*n* = 90), during the period March 2018–2019, were obtained from the Microbiology laboratory in El-Demerdash hospital, Faculty of Medicine, Ain Shams University (Cairo, Egypt). Identification was verified by detecting *bla*_OXA–51–like_ gene (intrinsic to *A. baumannii*) as described previously (El Far et al., 2019). *A. baumannii* ATCC 19606 was used as a positive control. All isolates were preserved in trypticase soy broth medium (TSB, Difco^®^, Franklin Lakes, NJ, United States) with 15% glycerol, at −80°C for subsequent uses (Abdel-Baky et al., 2017).

### Antibiogram of *A. baumannii* Isolates

Five antibiotics were selected with different mechanisms of action: doxycycline (Sedico Co., Giza, Egypt) and amikacin (Eipico Co., Tenth of Ramadan City, Egypt) interrupting protein synthesis, imipenem (Merck & Co., Kenilworth, NJ, United States) inhibiting cell wall synthesis, levofloxacin (Sedico Co., Giza, Egypt) inhibiting cell division, and colistin (Sedico Co., Giza, Egypt) disrupting the outer cell membrane. Microbroth dilution method was used to determine the minimum inhibitory concentrations (MICs) according to the Clinical and Laboratory Standards Institute (CLSI) guidelines (CLSI, 2018). Negative control wells contained only culture media to ensure sterility, while positive controls were inoculated with the organism, and *Escherichia coli* ATCC 25922 was used as a reference strain. Following a 24-h incubation at 37°C, 30-μl aliquots of 0.015% resazurin solution (Thermo Fisher Scientific, Waltham, MA, United States) were added to each well and further incubated for 2 h. Change in resazurin color from blue to pink indicates bacterial growth (Elshikh et al., 2016). The susceptibility patterns of the isolates were interpreted according to the CLSI M07-A11 protocol (CLSI, 2019).

### Microtiter Plate Biofilm Formation Assay

Biofilm formation was screened as described by Lee et al. (2008). In brief, overnight cultures in trypticase soya broth (Hi-Media, Mumbai, India) were used to prepare inoculum equivalent to 0.5 McFarland standard adjusted spectrophotometrically at A600, then diluted 1:20 in trypticase soya broth. Aliquots (200 μl) of each bacterial suspension were dispensed in three wells of a 96-well flat-bottomed microtiter plate (Corning, Corning, NY, United States) and incubated for 24 h at 37°C. After incubation, the bacterial suspensions were carefully aspirated; then, wells were (i) washed with 200 μl of phosphate-buffered saline (PBS) to remove planktonic cells, (ii) decanted and air dried for 15 min, and (iii) stained with 200 μl of 0.1% v/v crystal violet solution and left to stand for 15 min. Then, each well was washed with PBS to remove excess stain. Finally, the stained wells were solubilized with 200 μl of 33% v/v acetic acid and incubated at 37°C for 15 min, and the optical density was measured at 630 nm using microtiter plate reader (ELx800, Biotek, Winooski, VT, United States). Uninoculated wells served as negative controls, and *A. baumannii* ATCC19606 was used as a positive control for the biofilm formation. Results were recorded as means of absorbance readings from triplicate wells. As described previously (Lee et al., 2008), the *OD* cutoff value (*OD*_c_) for biofilm formation was defined as three standard deviations above the mean absorbance of the inoculum free negative control (*OD*_c_ = *OD*_avg. of negative control_ + 3 standard deviation (SD) of negative control). Accordingly, isolates were classified as non-biofilm formers when *OD* ≤ *OD*_c_, weak biofilm formers when *OD*_c_ < *OD* ≤ (2 × *OD*_c_), moderate biofilm formers when 2 × *OD*_c_< *OD* ≤ (4 × *OD*_c_), and strong biofilm formers when *OD* > (4 × *OD*_c_).

### PCR Amplification of β-Lactamase and Biofilm-Related Genes

Genomic DNA was extracted using GeneJet^TM^ genomic DNA extraction kit (Thermo Fisher Scientific, Waltham, MA, United States) according to the manufacturer’s instructions and stored at −20°C for future use. PCR was performed using previously described primers (Thermo Fisher Scientific, Waltham, MA, United States) to amplify *β*-lactamase encoding genes from Ambler class A, *bla*_PER–1_ (Yang et al., 2019); class B metallo-*β*-lactamases (MBLs), *bla*_VIM_ (Ellington et al., 2007), *bla*_NDM_ (Poirel et al., 2011); and class D oxacillinases, *bla*_OXA–23–like_, *bla*_OXA–24–like_ (Asadollahi et al., 2012), and *bla*_OXA–48–like_ (Poirel et al., 2011). Amplification conditions for class D oxacillinases genes were initial denaturation at 94°C for 5 min; 35 cycles of denaturation at 94°C for 30 s, annealing at 54°C for 30 s, and elongation at 72°C for 30 s; then a final extension at 72°C for 10 min. Similar conditions were used to amplify *bla*_PER–1,_ but the annealing temperature was set at 50°C and elongation at 72°C for 60 s. As for class B MBLs genes, an annealing at 58°C touchdown 52°C was implemented in a TECHNE thermocycler (Bibby Scientific Ltd., Staffordshire, United Kingdom). PCR assays for the detection of biofilm-related genes (*bap*, *csu*E, and *omp*A) were performed by a set of primers described in a previous study (Yang et al., 2019). Conditions of the PCR involved initial denaturation at 94°C for 5 min, followed by 35 cycles of denaturation at 94°C for 60 s, an annealing temperature at 57°C for 60 s, and elongation at 72°C for 60 s, and a final extension at 72°C for 10 min. Gel electrophoresis was carried out using the ADVANCE electrophoresis system (Mupid-exu, Tokyo, Japan) at 5 V/cm on 1.5% agarose gel, stained with 0.5 μg/ml ethidium bromide, and visualized under ultraviolet light.

### Antibacterial Activities of Cinnamic and Gallic Acids

First, cinnamic and gallic acids (LOBA Chemie, Boisar, India) stock solutions were prepared (Daneshfar et al., 2008; Bradley et al., 2015). In brief, 1.5 g of cinnamic acid was dissolved in 10 ml dimethyl sulfoxide (DMSO); then, distilled water was added to 100 ml, sonicated on water bath for 2 h at 80°C, and 0.1 N NaOH was added dropwise till complete solubilization, then distilled water was added to the final volume (200 ml). For gallic acid, 4 g was dissolved in 150 ml distilled water, sonicated on water bath for 30 min; then distilled water was added to the final volume (200 ml). Next, 100 μl of cation adjusted Mueller Hinton broth CAMHB (Hi-Media, Mumbai, India) was dispensed in each well of a 96-well microtiter plate. Then, twofold serial dilutions of cinnamic or gallic acid solutions were prepared in the culture media at a final volume of 100 μl. Afterward, the wells were inoculated with 100 μl of an overnight culture of the test isolate adjusted to 0.5 McFarland standard, and plates were incubated at 37°C for 18–24 h (Bardbari et al., 2018). The positive control wells were the bacteria in CAMHB and the used solvent without cinnamic nor gallic acids. While wells without bacterial inoculum served as negative controls. The MICs of cinnamic and gallic acids were recorded as the lowest concentrations that totally inhibit visible bacterial growth.

### Antibiofilm Activities of Cinnamic and Gallic Acids at Sub-Inhibitory Concentrations

Initially, 200 μl of 0.5 McFarland bacterial Suspensions were distributed in 96-well polystyrene microtiter plates. After that, 20 μl cinnamic or gallic acid solutions were added to the wells at concentrations equivalent to 1/2 and 1/4 MICs, and the plates were incubated for 24 h at 37°C. Plates were washed twice with phosphate-buffered saline, stained with crystal violet solution 0.1% v/v, and then the dye was resolubilized with 33% v/v acetic acid. The optical density of the wells was recorded at 630 nm using a microtiter plate reader (ELx800, Biotek, Winooski, VT, United States) (Tutar et al., 2016). Each assay was performed in triplicates, and wells free of dissolved cinnamic and gallic acids represented the positive controls for biofilm formation. The percentage of biofilm reduction (%) was calculated as [(*Ac*−*As*)/*Ac*×100], where “Ac” is the *OD*_630_ value of the positive control wells, and “As” is the *OD*_630_ value of the cinnamic or gallic acids treated sample wells (Shao et al., 2015).

### Scanning Electron Microscopy Analysis of Antibiofilm Activities of Cinnamic and Gallic Acids

A strong biofilm former *A. baumannii* isolate was selected to visualize the effect of cinnamic and gallic acids on its associated biofilm. First, 500 μl of the bacterial suspension (10^8^ CFU/ml) was dispensed into the wells of a 24-well plate enclosing a round glass cover slip (13 mm, Menzel Gläser, Braunschweig, Germany). For treated wells, sub-MICs of cinnamic or gallic acids (500 μl) were added and incubated for 24 h at 37°C. Afterward, the formed biofilms on the coverslips were fixed in 2.5% glutaraldehyde for 30 min at room temperature in 0.1 M cacodylate buffer (pH 7.2). Subsequent post-fixation with osmium tetroxide and dehydration for 15 min with an ethanol gradient including 30, 50, 70, 90, and 100% v/v. The fixed biofilms were then coated with gold and examined with scanning electron microscope (JEOL JSM-6390LV, Tokyo, Japan) to compare treated isolates to untreated ones as a reference (Lemos et al., 2018).

### Growth Rate Analysis

The growth rates of 10 strong biofilm formers, 10 weak biofilm formers, and ATCC19606 were measured. For each strain, 20 μl of an overnight culture adjusted to 0.5 McFarland standard were added to 180 μl tryptic soy broth (TSB) in 96-well plates and incubated at 37°C for 24 h. Bacterial growth was monitored by measuring the *OD*_600_ in a microtiter plate reader (ELx800, Biotek, Winooski, VT, United States) every 4 h until 48 h. Results were recorded as means of absorbance readings from triplicate wells. In addition, the growth inhibitory activity of subinhibitory concentrations (1/2 and 1/4 MICs) of cinnamic and gallic acids was determined against these isolates. All determinations were performed as triplicates using untreated growth controls (Huang et al., 2020; Li et al., 2021).

### Statistical Analysis

All analyses were carried out using R statistical platform^1^ in R-studio version 1.4.1106. Several R-packages were used in data analysis and visualization including *readxl*, *ggplot2*, *polycor*, and *ggdendro.* In quantitative variables, normality assumption was tested using chi-squared goodness-of-fit test. For normally distributed data, *t*-test and ANOVA were used to compare the means of two groups and multiple groups, respectively. Kruskal–Wallis (KW) test was used to compare the medians for non-normally distributed data. Mann–Whitney and Tukey’s honestly significant difference (HSD) tests were applied as *post-hoc* tests using Bonferroni correction method for multiple comparisons in the Kruskal–Wallis and ANOVA tests, respectively. Fisher’s exact (FE) test of independence was employed to analyze the associations between nominal variables. Moreover, the correlation between these variables was performed using the Spearman’s rank correlation, and strength of the association was expressed as Spearman’s correlation coefficient (*r*_s_) between −1 and +1. For all statistical analyses, *p* < 0.05 were considered statistically significant.

### Ethical Approval

All experiments and study protocols complied with relevant guidelines, regulations, and standards of the ethical committee of Faculty of Pharmacy, Ahram Canadian University, and international ethical guidelines for biomedical research. Since the collected *A. baumannii* isolates were obtained from biospecimen repositories at the Microbiology Laboratory in El-Demerdash Hospital, there was no direct or indirect contact with any patient, and consequently, no informed consent was required.

## Results

### Minimum Inhibitory Concentration Determination and Resistance Profiles

**FIGURE 1:**
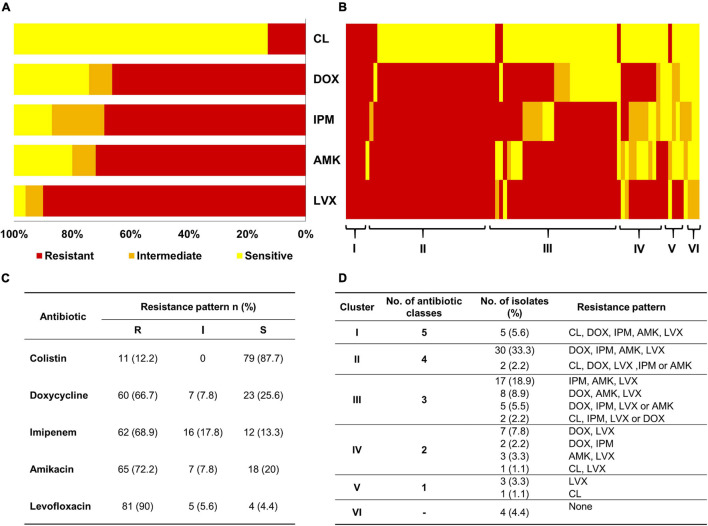
Sensitivity of *A. baumannii* isolates (*n* = 90) to different antibiotics as analyzed by MIC. **(A)** Stacked bar chart summarizing the resistance phenotypes of the 90 *A. baumannii* isolates to five antibiotics (y-axis). CL, colistin; DOX, doxycycline; IPM, imipenem; AMK, amikacin; LVX, levofloxacin. **(B)** Heatmap demonstrating the resistance pattern of each isolate as per the colors in the figure key (red, resistant; orange, intermediate; and yellow, sensitive). Isolates on the x-axis are sorted into six clusters footnoted on the heatmap. **(C)** Table summarizing the interpretation of MIC tests according to CLSI (2019). **(D)** Tabular representation of the detailed resistance patterns of the six clusters shown in Panel **(B)**.

Among the tested *A. baumannii* isolates (*n* = 90), resistance to levofloxacin (*n* = 81; 90%) was the most common, followed by resistance to amikacin (*n* = 65; 72.2%), imipenem (*n* = 62; 68.9%), doxycycline (*n* = 60; 66.7%), and colistin (*n* = 11; 12.2%), as shown in Figures 1A,C. For additional exploration, we clustered the isolates based on their antibiotic susceptibility patterns, and the resultant clusters are presented as a heat map (Figure 1B). The details of the six clusters are presented in Figure 1D. Notably, clusters 1–3 corresponded to 76.6% of the isolates revealing high prevalence of multiple drug resistance species. Only 4 out of 90 isolates were susceptible to the tested antibiotics exemplifying the high resistance rates detected in such isolates. High resistance level is usually described as having an MIC 16-fold or more the reported breakpoint (Mouton et al., 2018; Zhu et al., 2021). Accordingly MICs can be described as high because all the isolates have shown high resistance level against levofloxacin (*n* = 81/81); three quarters have shown high resistance level against amikacin (*n* = 49/65), 63% against doxycycline (*n* = 38/60), 55% against colistin (*n* = 6/11), and 34% against imipenem (*n* = 21/62). This is further demonstrated in the high MIC50 and MIC90 values of almost all the examined antibiotics (Table 1).

**TABLE 1:** Distribution of *Acinetobacter baumannii* isolates (*n* = 90) according to minimum inhibitory concentration (MIC) of the tested antimicrobial agents.

Antibiotic	Minimum inhibitory concentration (μg/ml)													
	<2	2	4	8	16	32	64	128	256	512	1024	>1024	MIC50	MIC90
Colistin	39	40	0	2	3	0	2	1	0	2	0	1	2	8
Imipenem	3	9	16	4	10	16	11	12	0	2	6	1	32	256
Doxycycline	0	8	15	7	7	11	4	1	9	8	20	0	32	1024
Amikacin	0	0	1	3	14	7	3	2	2	9	0	49	>1024	>1024
Levofloxacin	0	4	5	3	8	28	11	28	3	0	0	0	32	128

### Coexistence of Antimicrobial Resistance

**FIGURE 2:**
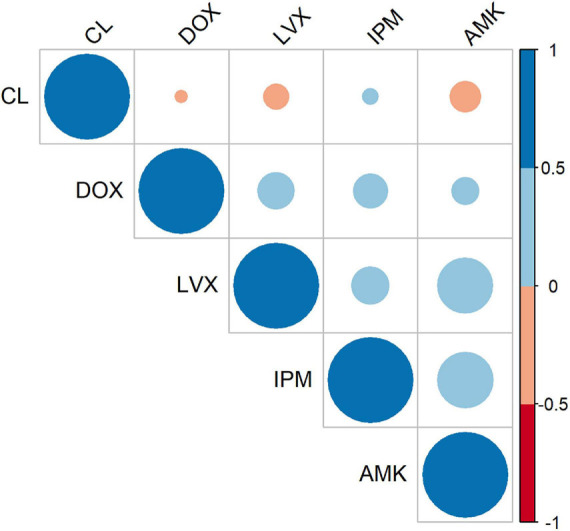
Antibiotic–antibiotic correlations. Correlogram representing correlation coefficients between each pair of antibiotics according to the patterns of susceptibility of the *A. baumannii* 90 isolates. The color intensity represents Spearman’s rank correlation coefficient (*r*s) value (blue tones are positive correlations, and red tones are negative correlations).

To evaluate the co-occurrence of resistance among various antibiotics, we built a correlation matrix between the tested agents employing the susceptibility patterns of the 90 isolates. As illustrated in Figure 2, positive and negative correlations were observed depending on the groups of antibiotics assessed. This analysis indicated that imipenem (IPM), amikacin (AMK), and levofloxacin (LVX) displayed the strongest correlations. Precisely, the Spearman’s correlation coefficient (*r*_s_) between AMK and IPM was 0.424 (*FE* test *p* = 0.00003) and between AMK and LVX was 0.423 (*FE* test *p* = 0.00003). These results are in accordance with the recorded resistance patterns (Figure 1D) in which coresistance of AMK and IPM was detected in 54 isolates (60%), while resistance to both AMK and LVX was documented in 66 isolates (73%).

### Strength of Biofilm-Formation by *A. baumannii* Isolates and Growth Rate Analysis

Effectively, the crystal violet biofilm assay differentiated *A. baumannii* isolates (*n* = 90) into strong, moderate, and weak biofilm formers. In particular, 11 isolates (12.2%) were categorized as weak biofilm formers, 48 (53.3%) were moderate, and 31 (34.5%) had strong biofilm-forming abilities. No significant differences (*p* > 0.05) in growth rates of the strong and weak biofilm formers were observed (Supplementary Table 1), indicating that the difference in biofilm formation was not due to the growth rate.

### Relationship Between Antibiotic Susceptibility and Biofilm-Forming Ability

The biofilm-forming abilities (strong, moderate, and weak) of *A. baumannii* isolates were determined among different antibiotic-resistance clusters (Table 2). Generally, a statistically significant association existed between clusters of isolates and intensity of biofilm (*FE* test *p* = 0.03). For instance, strong biofilm formers were enriched in clusters I, II, and III with the respective percentages 9.7, 41.9, and 12.9%. A similar tendency was observed for moderate biofilm formers with the percentages 4.2, 31.1, and 47.9%. Since clusters I–III are MDR isolates, the differences between multidrug-resistant isolates and susceptible ones, based on their biofilm formation, were evaluated, and a significant association was attained (*FE* test *p* = 0.042). To analyze whether the biofilm-forming ability is correlated with any of the tested antibiotics, we constructed a contingency table between the variables under consideration (Supplementary Table 2).

**TABLE 2:** Distribution of biofilm forming-abilities of *Acinetobacter baumannii* isolates among different antibiotic-resistance clusters.

Cluster	Biofilm formation *n* (% column)		
(No. of antibiotic	Strong	Moderate	Weak
classes)	(*n* = 31)	(*n = 48*)	(*n* = 11)
**I (5)**	3 (9.7%)	2 (4.2%)	0 (0%)
**II (4)**	13 (41.9%)	15 (31.3%)	4 (36.4%)
**III (3)**	4 (12.9%)	23 (47.9%)	5 (45.5%)
**IV (2)**	5 (16.1%)	7 (14.6%)	1 (9.1%)
**V (1)**	3 (9.7%)	1 (2.1%)	0 (0%)
**VI (0)**	3 (9.7%)	0 (0%)	1 (9.1%)

### Antibiotic-Resistance Correlations of Multidrug-Resistant *A. baumannii* Isolates

Out of the 90 screened *A. baumannii* isolates, 30 MDR isolates were selected for further study. None of the tested isolates were susceptible to levofloxacin, and over 90% exhibited resistance to imipenem, doxycycline, and amikacin. On the other hand, only six (20%) isolates displayed reduced susceptibility to colistin. A positive strong correlation between DOX and AMK resistance was observed (*r*_s_ = 0.557, *FE* test *p* = 0.001). However, a moderate negative correlation between CL and AMK (*r*_s_ = −0.37, *FE* test *p* = 0.043) was found.

### Molecular Detection of β-Lactamase-Encoding Genes and Genotypic–Phenotypic Correlations

Results of the detected *β-*lactamase-encoding genes and the corresponding imipenem MIC distributions for the 30 MDR *A. baumannii* are shown in Table 3. The results revealed almost equal occurrence of ambler class B MBLs (*n* = 13), ambler class D serine *β*-lactamases (*n* = 12), and class A *β*-lactamases (*n* = 14). Nevertheless, none of *A. baumannii* isolates harbored *bla*_OXA–24–like_ and *bla*_OXA–48–like_ genes. Interestingly, a strong negative correlation was observed between MBLs and serine *β*-lactamases genes (*r*_s_ = −0.714, *FE* test *p* = 0.0001). On the contrary, *bla*_VIM_ gene positively correlated with *bla*_NDM_ (*r*_s_ = 0.398, *FE* test *p* = 0.029). Furthermore, the effects of different genotypes on imipenem resistance of the isolates were assessed. A Kruskal–Wallis test showed that MBLs and class A *β*-lactamases genes significantly affect the imipenem MICs, *H*(2) = 6.0504, *p* = 0.0485. The difference in imipenem MICs between a genotype of a single MBL and a genotype of MBL combined with class A *β*-lactamases was statistically significant (*post-hoc* Mann–Whitney test, *p* = 0.02668). On the other hand, the difference in imipenem MICs was not significant among genotypes belonging to class D and class A *β*-lactamases (*p* > 0.05). Moreover, isolates harboring resistance genes were enriched in strong and moderate biofilm formers (Supplementary Table 3), but only *bla*_VIM_ and *bla*_OXA–23_ genes were significantly associated with the biofilm-forming ability (*FE* test, *p* = 0.0347).

**TABLE 3:** Prevalence of *β-*lactamase-encoding genes with the corresponding imipenem minimum inhibitory concentration (MIC) distributions for 30 isolates of *Acinetobacter baumannii.*

Genotype	No. of isolates (%)	Imipenem MIC (μg/ml)						Median
		16	32	64	128	256	512	
**Ambler class B and A *β*-lactamases**								
*bla*_NDM_ or *bla*_VIM_	6 (22.22)	–	1	–	1	2	2	256
*bla*_VIM_ + *bla*_NDM_ or *bla*_PER–1_	5 (18.52)	2	1	1	1	–	–	32
*bla*_VIM_ + *bla*_NDM_ + *bla*_PER–1_	2 (7.41)	–	–	–	1	–	1	320
Kruskal–Wallis test *H*(2) = 6.0504, *p* = 0.0485								
**Ambler class D and A *β*-lactamases**								
*bla*_PER–1_	2 (7.41)	–	–	2	–	–	–	64
*bla*_OXA–23_	4 (14.81)	–	1	1	1	1	–	96
*bla*_OXA–23_ + *bla*_PER–1_	8 (29.63)	1	–	2	1	4	–	192
Kruskal–Wallis test *H*(2) = 1.3247, *p* = 0.5156								

### Correlation Between Biofilm-Formation Ability and Detection of Biofilm-Related Genes

**FIGURE 3:**
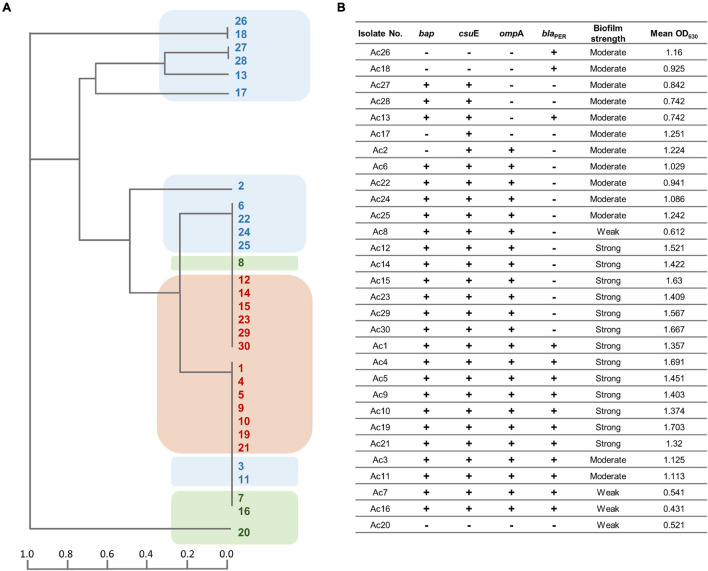
**(A)** Dendrogram signifying the clustering relatedness of 30 MDR *A. baumannii* isolates based on their PCR results for biofilm-related genes. The clusters are highlighted according to their biofilm strength (brown, strong; blue, moderate; green, weak biofilm forming). **(B)** Tabular presentation of the PCR screening results of biofilm associated genes and corresponding mean *OD*_630_ values.

The incidence of *bla*_PER–1_, *bap*, *omp*A, and *csu*E genes amid the isolates was 46.7, 83.8, 76.7, and 90%, respectively. The tested isolates were classified based on the patterns of genes distribution using the unweighted pair group method with arithmetic mean (UPGMA) algorithm for hierarchical clustering (Figure 3). As shown in the dendrogram (Figure 3A), strong biofilm-forming isolates occupied a single major clade. However, a few moderate and weak biofilm formers were clustered in close vicinity of the strong ones. After analyzing the association between the biofilm-forming ability and biofilm-related genes, a statistically significant difference was found with *omp*A (*FE* test *p* = 0.0125), *omp*A + *bap* (*FE* test *p* = 0.0122), *omp*A + *csu*E (*FE* test *p* = 0.02), and *omp*A + *bap* + *csu*E (*FE* test *p* = 0.02). Furthermore, a strong positive correlation existed between *csu*E along with both *bap* and *omp*A, genes (*r*_s_ = 0.745 and 0.604, respectively, *FE* test *p* < 0.05). Similarly, *bap* positively correlated with *omp*A (*r*_s_ = 0.599, *FE* test *p* = 0.00047). Intriguingly, the concurrence of either *omp*A and *bap* or *omp*A and *csu*E had equivalent predictive value of strong biofilm formation (100%) as the simultaneous detection of the three genes *omp*A, *bap*, and *csu*E (Table 4).

**TABLE 4:** Genotype–phenotype associations of biofilm-related genes with biofilm formation.

	Biofilm formation *n*			Fisher’s
Biofilm-related genes	(% column)			exact test
	Strong	Moderate	Weak	
***Omp*A** + ***bap***				*p* = 0.012
Both negative	0 (0%)	3 (23%)	1 (25%)	
Only one positive	0 (0%)	4 (31%)	0 (0%)	
Both positive	13 (100%)	6 (46%)	3 (75%)	
***Omp*A** + ***csu*E**				*p* = 0.02
Both negative	0 (0%)	2 (15.5%)	1 (25%)	
Only one positive	0 (0%)	4 (31%)	0 (0%)	
Both positive	13 (100%)	7 (53.5%)	3 (75%)	
***Omp*A** + ***bap* + *csu*E**				*p* = 0.02
All negative	0 (0%)	2 (15.5%)	1 (25%)	
Only one positive	0 (0%)	1 (7.5%)	0 (0%)	
Only two positive	0 (0%)	4 (31%)	0 (0%)	
All positive	13 (100%)	6 (46%)	3 (75%)	
***Omp*A** + ***bap* + *bla*_PER–1_**				*p* = 0.027
All negative	0 (0%)	1 (7.5%)	1 (25%)	
Only one Positive	0 (0%)	5 (38.5%)	0 (0%)	
Only two Positive	6 (47%)	5 (38.5%)	1 (25%)	
All Positive	7 (53%)	2 (15.5%)	2 (50%)	
***Omp*A** + ***csu*E + *bla*_PER–1_**				*p* = 0.015
All negative	0 (0%)	0 (0%)	1 (25%)	
Only one positive	0 (0%)	5 (38.5%)	0 (0%)	
Only two positive	6 (47%)	6 (46%)	1 (25%)	
All positive	7 (53%)	2 (15.5%)	2 (50%)	

### Antimicrobial Activity of Gallic and Cinnamic Acids

**FIGURE 4:**
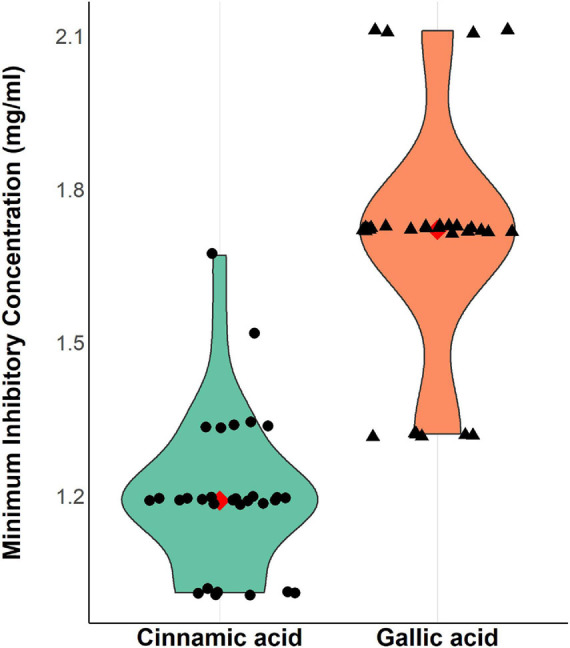
Violin plots showing minimum inhibitory concentrations (mg/ml) of cinnamic and gallic acids against 30 MDR *A. baumannii* isolates. A strip chart displaying individual isolates was overlaid to illustrate the real dispersal of MIC values. The filled red diamond signifies the MIC mean value (1.2, 1.67 mg/ml for cinnamic and gallic acids, respectively) allowing better assessment of the data. Plot was created by R (“ggplot2”) package.

The results revealed a promising antimicrobial activity against *A*. *baumannii*; MICs of cinnamic acid ranged from 1.01 to 1.67 mg/ml, while those of gallic acid ranged from 1.32 to 2.11 mg/ml (Figure 4). Cinnamic acid (mean MICs = 1.2 mg/ml) displayed higher antibacterial activity against *A*. *baumannii* as compared to that of gallic acid (mean MICs = 1.65 mg/ml) (*t*-test = −10.947, *p* = 8.147 × 10^–12^).

### The Anti-biofilm Activities of Gallic and Cinnamic Acids

**FIGURE 5:**
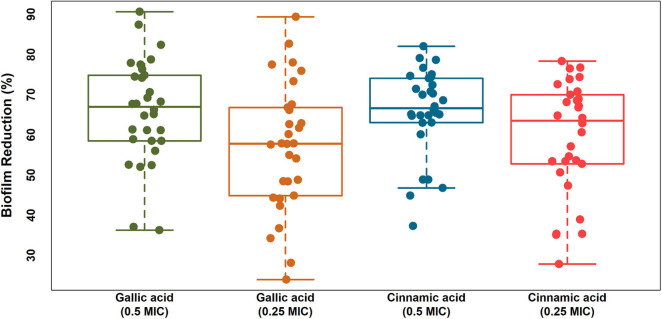
Boxplots displaying biofilm reduction (%) caused by subinhibitory concentrations (1/2 and 1/4 MICs) of cinnamic and gallic acids. A strip chart displaying individual isolates was overlaid to illustrate the real dispersal of reduction percentages. The means were significantly different [ANOVA *F*(3), *p* = 0.0198]. Plot was created by R (“ggplot2”) package.

**FIGURE 6:**
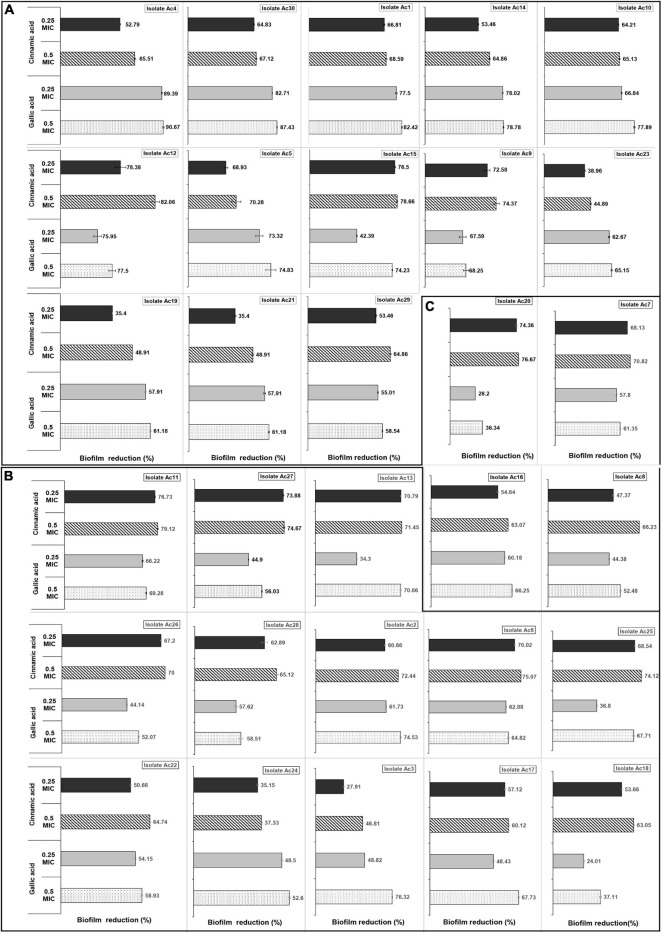
Inhibitory effect of cinnamic and gallic acids on biofilm formation of each of the 30 MDR *A. baumannii* isolates. Clustered bar charts demonstrate biofilm reduction (%) following treatment with subinhibitory concentrations of cinnamic acid 1/2 MICs (stripped bars), 1/4 MICs (black bars), and gallic acid 1/2 MICs (dotted bars), and 1/4 MICs (gray bars) on **(A)** strong, **(B)** moderate, and **(C)** weak biofilm formers.

**FIGURE 7:**
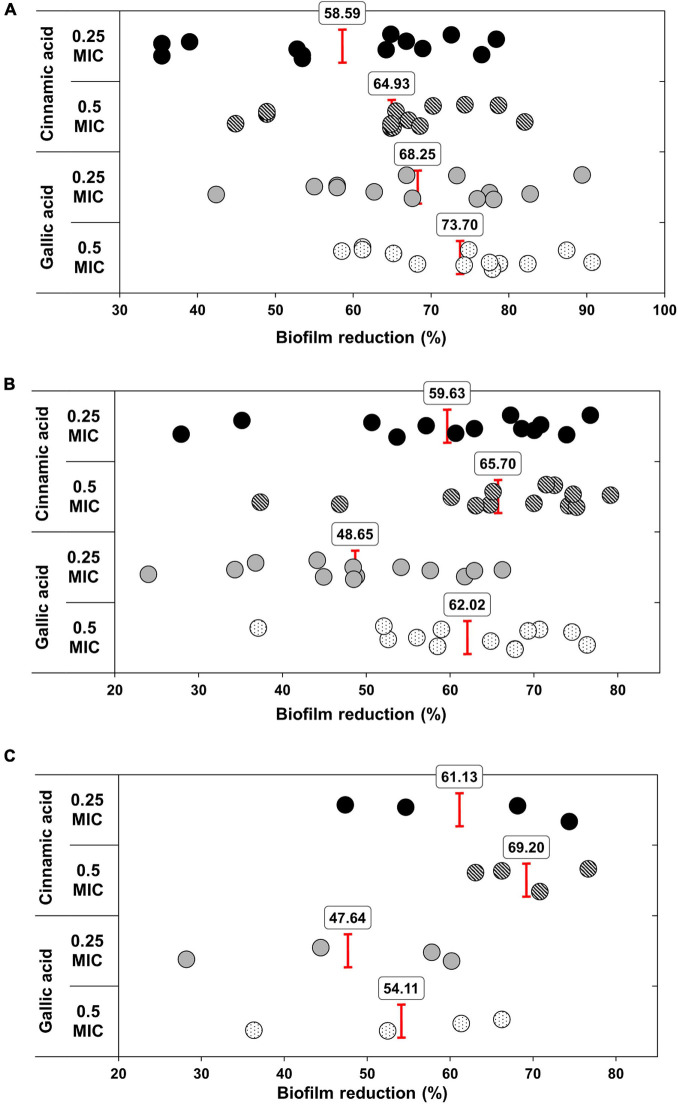
Antibiofilm activities of sub-MICs of cinnamic and gallic acids. Scatter plot representing biofilm reduction (%) for each sample. Mean reduction percentages of 1/2 and 1/4 MICs cinnamic and gallic acids are illustrated against **(A)** strong biofilm formers [ANOVA *F*(3) = 6.4152, *p* < 0.05], **(B)** moderate biofilm formers [ANOVA *F*(3) = 6.5257, *p* < 0.05], and **(C)** weak biofilm formers [ANOVA *F*(3) = 2.3125, *p* > 0.05].

Notably, both gallic and cinnamic acids resulted in significant reductions in the biofilm formation associated with *A. baumannii* isolates (Figure 5). The superlative antibiofilm activity was attained at ½ MIC, which led to inhibition percentages reaching 82 and 91% for cinnamic and gallic acids, respectively. A statistically significant difference in the effect between the subinhibitory concentrations of cinnamic and gallic acids was observed by one-way ANOVA [*F*(3) = 3.415, *p* = 0.0198]. For a deeper investigation of the antibiofilm activities of both acids, we reanalyzed their effects after dissecting the isolates into strong, moderate, and weak biofilm formers (Figure 6). Regarding strong biofilm formers (Figure 6A), subinhibitory concentrations of gallic acid displayed enhanced effect against all isolates except Ac9, Ac12, Ac15, and Ac29 (*r*s = 0.598, *p* < 0.05). Conversely, subinhibitory concentrations of cinnamic acid showed greater activity on all moderate biofilm formers, except Ac2, Ac3, Ac17, Ac22, and Ac24 (Figure 6B). A similar trend was observed in weak biofilm formers (Figure 6C). Statistical analysis showed that the type and concentration of the tested agent significantly affected reduction in biofilm formation among strong biofilm formers [*F*(3, 36) = 6.4152, *p* < 0.05]. As shown in Figure 7A, a higher antibiofilm activity of the subinhibitory concentrations (1/2 and 1/4 MIC) of gallic acid (mean reduction = 73.70 and 68.25%, respectively) was observed as compared to cinnamic acid (mean reduction = 64.93 and 58.59%, respectively), whereas analysis of moderate biofilm forming isolates proved enhanced activity of the subinhibitory concentrations (1/2 and 1/4 MIC) of cinnamic acid (mean reduction = 65.70 and 59.63%, respectively) versus gallic acid (mean reduction = 62.02 and 48.65%, respectively) [*F*(3, 36) = 6.5257, *p* < 0.05] (Figure 7B). Concerning weak biofilm formers, despite the large discrepancies between the effect of subinhibitory concentrations (1/2 and 1/4 MIC) of cinnamic acid (mean reduction = 69.20 and 61.13%, respectively) versus gallic acid (mean reduction = 54.11 and 47.64%, respectively), no statistically significant difference was detected (*p* > 0.05), which may be attributed to small number of isolates in this group (Figure 7C). Noteworthy, the effect of subinhibitory concentrations (1/2 and 1/4 MICs) of cinnamic and gallic acids on the growth of the tested *A. baumannii* isolates are shown in figures (Supplementary Figures 1, 2). Despite the slight growth pattern differences between the control and some of the treated isolates, sub-MICs of cinnamic and gallic acids generally did not affect the viability of the tested strains.

### Impact of Resistance Profiles and Biofilm-Related Genes on Biofilm Susceptibility to Gallic and Cinnamic Acids

The only statistically significant positive correlation between the antibiotic MICs and the percentages of biofilm reduction was shown with levofloxacin (*r*_s_ = 0.45, *FE* test *p* = 0.0115). As for the plausible effect of biofilm-related genes, including *bap*, *csu*E, *omp*A, and *bla*_PER–1_, on the documented percentages of biofilm reduction, a statistically significant difference was obtained using sub-MICs of gallic acid [*F*(7) = 3.293, *p* = 0.015] within the following genotype pairs, (*bap* + *csu*E + *omp*A + *bla*_PER–1_ and *bla*_PER–1_) (means = 71.8 vs. 44.6%) and (*bap* + *csu*E + *omp*A + *bla*_PER–1_ and no genes) (means = 71.8 vs. 36.34%).

### SEM Analysis of Antibiofilm Activities of Cinnamic and Gallic Acids

**FIGURE 8:**
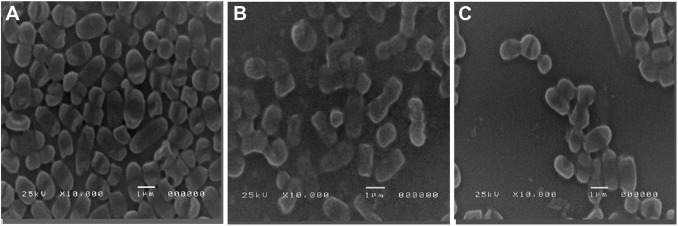
Scanning electron microscopy images of **(A)** untreated strong biofilm-former *A. baumannii* isolate, after **(B)** a 24-h treatment with sub-MIC cinnamic acid, and **(C)** a 24-h treatment with sub-MIC gallic acid. Magnification 10,000×.

SEM images of the untreated control biofilm showed a dense layer of colonized cells (Figure 8A), whereas a conspicuous inhibition of *A. baumannii* biofilm formation was noticed in the treated biofilm in which cells were individually visualized (Figures 8B,C). It is noteworthy that gallic acid possessed significantly higher activity (Figure 8C) compared to cinnamic acid (Figure 8B), consistent with our findings using microtiter plate assay.

## Discussion

*A. baumannii* infections pose a serious concern triggered by the elevated rates of multidrug resistance (Eze et al., 2018). This problem is further aggravated by its ability to form biofilms (Yang et al., 2019). Antibiotic resistance in biofilms is estimated to be ∼1,000-fold higher than in planktonic cells, thus restricting available choices of effective antimicrobial treatments (Hall and Mah, 2017). In the present study, 76.6% (69/90) of our isolates were MDR displaying resistance patterns consistent with previous reports (Tchuinte et al., 2019; Araújo Lima et al., 2020). Our findings reveal higher levels of colistin resistance (12.2%) compared to recent studies from the region (Abdulzahra et al., 2018; Elham and Fawzia, 2019). This noticeable increase in colistin resistance is alarming because it is considered the last resort antibiotic for the treatment of severe infections caused by carbapenem resistant *A. baumannii* (Thet et al., 2020). Moreover, we found statistically significant correlations between resistance to AMK and to both IPM and LVX (*r*s = 0.424 and 0.423, respectively). Yu et al. (2007) previously reported a widespread occurrence of amikacin resistance in imipenem-resistant *A. baumannii* due to aminoglycoside resistance methyltransferase (*ArmA*). Similar correlations between IPM and LVX were recently described (El-Din et al., 2021), this may be attributed to the various impacts that resulted from a mutation at one or a few genetic loci (Adamus-Bialek et al., 2013). Our isolates exhibited much higher frequency (100%) of biofilm production as opposed to *A. baumannii* clinical isolates (70.1%) lately investigated from Egypt (Asaad et al., 2021), Iran (70.6%) (Ranjbar and Farahani, 2019), and China (54%) (Chen et al., 2020). Several studies linked high frequency of biofilm formation in *A. baumannii* with extended survival and resistance to external stresses such as limited nutrients and dehydration (Badave and Kulkarni, 2015; Sung, 2018). The nature of association between resistance profiles and biofilm formation in *A. baumannii* is somewhat controversial (Eze et al., 2018). According to some studies, biofilm formation is strongly associated with MDR strains than with susceptible ones (Babapour et al., 2016; Bardbari et al., 2017; Yang et al., 2019). On the other hand, an inverse relation between biofilm formation and antibiotic resistance has been lately documented (Qi et al., 2016; Shenkutie et al., 2020). The current study investigated the potential association between phenotypic–genotypic resistance profiles and the biofilm-forming ability of clinical *A. baumannii* isolates. In view of this, our results highlight a significant association between MDR and biofilm formation. Relevant finding of this association was proclaimed by researchers from Egypt (Asaad et al., 2021), India (Badave and Kulkarni, 2015), Bangladesh (Nahar et al., 2013), Iran (Ranjbar and Farahani, 2019), and Mexico (Bocanegra-Ibarias et al., 2015). Increased synthesis of exopolysaccharide in biofilm-forming *A. baumannii* probably creates a barrier to antibiotic penetration, hence development of resistance. Slow growth rate, variations in the cellular physiology of cells, and enhanced horizontal gene transfer within the biofilm communities may be another explanation for high resistance associated to biofilms (Lewis, 2001; Donlan and Costerton, 2002; Yang et al., 2019). Albeit not every resistance profile of our isolates was associated with stronger biofilm formation, a statistically significant correlation occurred with susceptibility to certain antibiotics. On the one hand, biofilm formation was associated (*p* < 0.05) with resistance to amikacin. A recent study (Yang et al., 2019) reported the association between resistance to aminoglycosides and biofilm formation. This might be explained by induced gene regulation offering a fitness advantage for such resistant strains to form biofilms (Qi et al., 2016). We also noted a significant relation between biofilm formation and levofloxacin resistance in agreement with other studies (Coyne et al., 2010; He et al., 2015) reporting overexpression of the *Ade*FGH efflux pump leading to high-level resistance to fluoroquinolone and increased biofilm formation.

Several Ambler class *β*-lactamase-encoding genes were detected, *bla*_OXA–23_ (40%), *bla*_VIM_ (36.6%), and *bla*_NDM_ (23.3%). While *bla*_OXA–51–like_ genes were present in all the isolates, *bla*_OXA–24_- and *bla*_OXA–48_-encoding genes were absent. Acquired *β*-lactamase-encoding genes, disseminating via mobile genetic elements, were concurrently present in 50% of the isolates, regrettably warning for further spread via horizontal gene transfer (Linciano et al., 2020). Although MBLs are usually coexpressed with serine *β*-lactamases (Meini et al., 2014), we conversely delineate a negative correlation between their encoding genes (*r*_s_ = −0.714, *p* < 0.05). Noteworthy, the coexistence of *bla*_PER–1_ with multiple MBL encoding genes was significantly associated with high imipenem MICs. Likewise, researchers from Iran (Bagheri Josheghani et al., 2015) and Saudi Arabia (Aly et al., 2016) characterized the high prevalence of *bla*_PER–1_ gene among carbapenem-resistant *A. baumannii*.

Another goal of this study was to assess the relationships between biofilm formation and the presence of biofilm-related and antibiotic resistance genes in the isolates. For this purpose, we found a significant association between biofilm formation and the presence of either *bla*_OXA–23_ or *bla*_VIM_ genes (*p* < 0.05). Formerly, Azizi et al. (2015) reported that *A. baumannii* isolates with class-D OXA carbapenemases genes had strong biofilm capability. Several studies showed that *Pseudomonas aeruginosa* isolates harboring *bla*_VIM_ gene produced stronger biofilms (Heydari and Eftekhar, 2015). To the best of our knowledge, this is the first report of association between biofilm formation and *bla*_VIM_ gene in *A. baumannii.* This may represent a profound concern due to the likelihood of horizontal gene transfer between isolates closely located within biofilms (Abe et al., 2020; Uruén et al., 2021). Regarding the biofilm-related genes, our results agree with that of Zeighami et al. (2019), in which the most frequently detected gene was *csu*E (90%), followed by *bap* (83.8%), *omp*A (76.7%), and *bla*_PER–1_ (46.7%). In compliance with other investigators (Bardbari et al., 2017), no relationship between biofilm formation and production of *bla*_PER–1_ could be established. A conceivable rationalization for this could be that *bla*_PER–1_ gene might be associated with increased cell adhesiveness without promoting biofilm formation (Yang et al., 2019). In *A. baumannii* isolates, *omp*A is involved in resistance to antimicrobial agents, needed for attachment to eukaryotic cells, and partially contributes to biofilm formation (Yang et al., 2019). This investigation validated a significant association between the biofilm intensity and presence of *omp*A gene (*p* = 0.0125). Remarkably, we recorded a strong positive correlation between *omp*A and both *bap*, which is responsible for the expression of biofilm-associated proteins on the bacterial cell surfaces, and *csu*E, which mediates adhesion and biofilm formation. Moreover, a statistically significant association was detected between these genes and the biofilm-forming strength. Hence, these gene pairs are considered of great diagnostic value for the discrimination of strong biofilm-forming phenotype. Strikingly, three of the weak biofilm-forming isolates also had some biofilm-related genes, emphasizing the role of factors such as stress, nutrition, environment, and pathogen itself in biofilm induction and development (Al-Shamiri et al., 2021).

Taken together, the above analyses disclose the scale of the problem imposed by MDR *A. baumannii.* During the last decade, research studies on remedies with natural products were conducted to provide alternative antimicrobials for combating MDR isolates. For instance, cinnamic acid displayed noticeable antimicrobial effects against food-borne pathogens and standard and clinical isolates (Alves et al., 2013; Guzman, 2014; Zhang et al., 2019). Nonetheless, as far as we know, scarce data are available about its antimicrobial activity against MDR *A. baumannii.* Gallic acid is a phenolic acid with reported antibacterial activity, especially in combination with conventional antibiotics (Shao et al., 2015; Dos Santos et al., 2018; Farrag et al., 2019). Notably, the mean minimal effective dose of cinnamic acid (1.2 mg/ml) was significantly lower than that of gallic acid (1.67 mg/ml). This significant reduction in the antimicrobial activity of gallic acid has been attributed to increased number of hydroxyl group substitutions on the benzene ring compared to cinnamic acid (Sánchez-Maldonado et al., 2011; Zhang et al., 2019). One interesting observation from the present study was the efficiency of cinnamic and gallic acids in limiting the formation of biofilm associated with MDR *A. baumannii*. Former studies demonstrated that gallic acid inhibited biofilm formation in *Staphylococcus aureus* (Luís et al., 2014), *P. aeruginosa* (Zhang et al., 2020), *E. coli*, and *Streptococcus mutans* (Shao et al., 2015). Likewise, the antibiofilm effect of cinnamic acid derivatives was recognized in recent studies (Zhou et al., 2020). Various mechanisms have been proposed to justify the antibiofilm activities of phenolic compounds including gallic and cinnamic acid derivatives. They may exert their effects through cleavage of peptidoglycan present in the cell wall (Burcu Bali et al., 2019) and/or inhibition of N-acyl homo-serine lactones (AHLs)-mediated quorum sensing (Rajkumari et al., 2018; Zhang et al., 2020), coupled with the antioxidant power of these compounds, which prevents the formation of reactive oxygen species (ROS), and accordingly might obstruct the expression of key genes implicated in the regulation of biofilm formation (Ong et al., 2018). The SEM images reinforced the antibiofilm effect of gallic and cinnamic acids on the tested isolates. In their review about the most extensively used methods for biofilm visualization, Relucenti et al. (2021) considered SEM as the most efficient tool for comparative biofilm investigations, particularly when testing antibiofilm agents (Relucenti et al., 2021). Raorane et al. (2019) used SEM to investigate the antibiofilm effect of curcumin on *Candida albicans* and *A. baumannii* mixed biofilms and reported that SEM images of untreated biofilms showed large hyphae encasing *A*. *baumannii* cells; however, after treatment with 20 μg/ml curcumin, disintegration of biofilms was obvious, and *A*. *baumannii* cells could be clearly recognized (Raorane et al., 2019). Similarly, analysis of the obtained SEM images revealed that the treated *A. baumannii* biofilms showed distinct morphological changes compared to the untreated one. A dense matrix of intact cells was observed in the untreated biofilm, while treated ones displayed structural alterations to the matrix with visible reduction in the adherent cells along with damage to the cells. Remarkably, the damage in the structural integrity of the biofilms was more obvious in gallic-acid-treated biofilms compared to cinnamic-acid-treated ones.

## Conclusion

This work shed the light on the debatable multifactorial correlation between biofilm formation and resistance of *A. baumannii* clinical isolates. The results underlined a significant association between MDR and the biofilm-forming ability. Altogether, the presence of *bla*_VIM_ and *bla*_OXA–23_, along with the biofilm-related genes *omp*A, *bap*, and *csu*E influence the intensity of the formed biofilms. Exploring the relationship between antibiotic resistance and biofilm formation may unravel the complex dilemma of managing MDR pathogens, in general, and *A. baumannii*, in particular. Furthermore, this study provided a proof that cinnamic and gallic acids exhibited inhibitory and substantial antibiofilm activities against MDR *A. baumannii* isolates.

## Data Availability Statement

The original contributions presented in the study are included in the article/Supplementary Material, further inquiries can be directed to the corresponding author.

## Author Contributions

WE, NA, and NE conceived the objectives and designed the study protocol. NA and MS provided the isolates. MS and WK participated in methodology and experimental work. NA performed the statistical analysis, prepared tables and figures, and wrote the manuscript in its required format. MS, WK, and NA analyzed the data and interpreted the results. NA, NE, and MS drafted the manuscript. WE, NA, NE, and WK revised the manuscript prior to its submission. WE chiefly supervised the study and critically revised and edited the manuscript. All authors read and approved the final submitted version.

## Conflict of Interest

The authors declare that the research was conducted in the absence of any commercial or financial relationships that could be construed as a potential conflict of interest.

## Publisher’s Note

All claims expressed in this article are solely those of the authors and do not necessarily represent those of their affiliated organizations, or those of the publisher, the editors and the reviewers. Any product that may be evaluated in this article, or claim that may be made by its manufacturer, is not guaranteed or endorsed by the publisher.

## Footnotes

1
https://www.r-project.org
