# Supplementary material for: Multidrug Resistant Acinetobacter baumannii Biofilms: Evaluation of Phenotypic–Genotypic Association and Susceptibility to Cinnamic and Gallic Acids
Source: Front Microbiol. 2021 Sep 17;12:716627. doi: 10.3389/fmicb.2021.716627 (PMC8508616; doi:10.3389/fmicb.2021.716627)
Supplement: Supplementary file 1 [file Data_Sheet_1.docx]

**MDR *Acinetobacter* *baumannii* biofilms: Evaluation of phenotypic-genotypic association and susceptibility to cinnamic and gallic acids**

**Mahmoud M. Sherif^1^, Walid F. Elkhatib^2,3*^, Wafaa S. Khalaf^4^, Nooran S. Elleboudy^2^ and Neveen A. Abdelaziz^1^**

^1^Department of Microbiology and Immunology, Faculty of Pharmacy, Ahram Canadian University, Sixth of October City, Giza, Egypt

^2^Department of Microbiology and Immunology, Faculty of Pharmacy, Ain Shams University, Abbassia, Cairo, Egypt

^3^Department of Microbiology & Immunology, Faculty of Pharmacy, Galala University, New Galala city, Suez, Egypt

^4^Department of Microbiology and Immunology, Faculty of Pharmacy (Girls), Al-Azhar University, Nasr city, Cairo, Egypt

* **Corresponding Author:**

**Prof. Dr. Walid F. Elkhatib**

Postal address: Microbiology and Immunology Department, Faculty of Pharmacy, Ain Shams University, African Union Organization St., Abbassia, Cairo 11566, Egypt

Tel: +202-24051120, Fax: +202-24051107

Emails: walid-elkhatib@pharma.asu.edu.eg; [walid2005faisal@yahoo.com](mailto:walid2005faisal@yahoo.com)

ORCID <https://orcid.org/0000-0001-5815-3200>

**Supplementary Tables**

**Table S1 Growth rate analysis results of 10 strong biofilm formers and 10 weak biofilm formers *Acinetobacter* *baumannii* isolates recorded as absorbance readings**

| **Time (hr)** | **OD_600_ for strong biofilm formers** | | | | | | | | | | |  | | **OD_600_ for weak biofilm formers** | | | | | | | | | | | ***p-*value** | |
| --- | --- | --- | --- | --- | --- | --- | --- | --- | --- | --- | --- | --- | --- | --- | --- | --- | --- | --- | --- | --- | --- | --- | --- | --- | --- | --- |
|  | **S1** | **S2** | **S3** | **S4** | **S5** | **S6** | **S7** | **S8** | **S9** | **S10** |  | | **W1** | | **W2** | **W3** | **W4** | **W5** | **W6** | **W7** | **W8** | **W9** | **W10** |  | |  |
| **0** | 0.109 | 0.106 | 0.107 | 0.114 | 0.118 | 0.115 | 0.112 | 0.122 | 0.109 | 0.111 |  | | 0.132 | | 0.112 | 0.112 | 0.102 | 0.107 | 0.117 | 0.11 | 0.111 | 0.142 | 0.118 | 0.3454 | |  |
| **4** | 0.368 | 0.283 | 0.264 | 0.262 | 0.306 | 0.265 | 0.28 | 0.325 | 0.315 | 0.247 |  | | 0.267 | | 0.199 | 0.361 | 0.276 | 0.266 | 0.29 | 0.341 | 0.345 | 0.276 | 0.316 | 0.9101 | |  |
| **8** | 0.551 | 0.403 | 0.391 | 0.392 | 0.443 | 0.769 | 0.396 | 0.521 | 0.498 | 0.401 |  | | 0.468 | | 0.727 | 0.542 | 0.391 | 0.352 | 0.452 | 0.514 | 0.457 | 0.387 | 0.433 | 0.9345 | |  |
| **12** | 0.861 | 0.51 | 0.537 | 0.489 | 0.671 | 1.031 | 0.546 | 0.678 | 0.662 | 0.486 |  | | 0.643 | | 1.027 | 0.756 | 0.541 | 0.437 | 0.567 | 0.674 | 0.556 | 0.594 | 0.651 | 0.9740 | |  |
| **16** | 0.991 | 0.813 | 0.786 | 0.792 | 0.963 | 1.142 | 0.787 | 0.786 | 0.734 | 0.571 |  | | 0.768 | | 1.147 | 0.852 | 0.791 | 0.498 | 0.639 | 0.791 | 0.649 | 0.671 | 0.965 | 0.4477 | |  |
| **20** | 1.032 | 1.327 | 0.896 | 1.35 | 1.376 | 1.192 | 0.897 | 0.987 | 0.956 | 0.621 |  | | 0.946 | | 1.194 | 0.97 | 0.851 | 0.546 | 0.671 | 0.991 | 0.717 | 0.831 | 1.375 | 0.1763 | |  |
| **24** | 1.139 | 1.438 | 1.19 | 1.432 | 1.473 | 1.209 | 0.945 | 1.093 | 1.137 | 0.736 |  | | 1.103 | | 1.209 | 1.078 | 0.873 | 0.703 | 0.719 | 1.083 | 0.776 | 0.861 | 1.47 | 0.0884 | |  |
| **28** | 1.136 | 1.402 | 1.25 | 1.41 | 1.424 | 1.203 | 0.897 | 1.287 | 1.144 | 0.651 |  | | 1.275 | | 1.231 | 1.196 | 0.914 | 0.644 | 0.724 | 1.28 | 0.81 | 0.91 | 1.43 | 0.2461 | |  |
| **32** | 1.111 | 1.38 | 1.19 | 1.362 | 1.382 | 1.214 | 0.926 | 1.395 | 1.152 | 0.65 |  | | 1.241 | | 1.243 | 1.218 | 0.952 | 0.669 | 0.719 | 1.399 | 0.819 | 0.985 | 1.390 | 0.3363 | |  |
| **36** | 1.103 | 1.38 | 1.16 | 1.371 | 1.376 | 1.225 | 0.927 | 1.416 | 1.145 | 0.649 |  | | 1.235 | | 1.257 | 1.228 | 0.971 | 0.693 | 0.706 | 1.401 | 0.832 | 1.006 | 1.376 | 0.3688 | |  |
| **40** | 1.087 | 1.354 | 1.14 | 1.356 | 1.361 | 1.237 | 0.904 | 1.362 | 1.135 | 0.638 |  | | 1.233 | | 1.271 | 1.218 | 1.01 | 0.726 | 0.686 | 1.352 | 0.832 | 1.001 | 1.361 | 0.4325 | |  |
| **44** | 1.073 | 1.321 | 1.132 | 1.335 | 1.361 | 1.241 | 0.889 | 1.314 | 1.13 | 0.637 |  | | 1.197 | | 1.287 | 1.212 | 0.974 | 0.754 | 0.673 | 1.324 | 0.825 | 1.001 | 1.361 | 0.4535 | |  |
| **48** | 1.053 | 1.313 | 1.128 | 1.341 | 1.369 | 1.247 | 0.879 | 1.301 | 1.127 | 0.638 |  | | 1.19 | | 1.28 | 1.221 | 0.963 | 0.751 | 0.662 | 1.311 | 0.815 | 0.998 | 1.355 | 0.4428 | |  |

**Table S2** Distribution of biofilm formation of 90 *A.baumannii* isolates with various susceptibilities against five antibiotics

| **Susceptibility pattern** | **Biofilm formation *n* (% column)** | | |  | **Fisher's Exact Test^1^** |  |
| --- | --- | --- | --- | --- | --- | --- |
|  | **Strong** | **Moderate** | **Weak** |  |  |  |
|  | **(*n=*31)** | ***(n=48)*** | ***(n=11)*** |  |  |  |
| **Colistin** |  |  |  |  | *p-*value= 0.8104 |  |
| Resistant | 5 (16.1%) | 5 (10.4%) | 1 (9.1%) |  |  |  |
| Sensitive | 26 (83.9%) | 43 (89.6%) | 10 (90.9%) |  |  |  |
| **Imipenem** |  |  |  |  | *p-*value= 0.3997 |  |
| Resistant | 19 (61.3%) | 34 (70.8%) | 9 (81.8%) |  |  |  |
| Intermediate | 8 (25.8%) | 6 (12.5%) | 2 (18.2%) |  |  |  |
| Sensitive | 4 (12.9%) | 8 (16.7%) | 0 (0%) |  |  |  |
| **Doxycycline** |  |  |  |  | *p-*value= 0.4511 |  |
| Resistant | 23 (74.2%) | 30 (62.5%) | 7 (63.6%) |  |  |  |
| Intermediate | 2 (6.5%) | 3 (6.3%) | 2 (18.2%) |  |  |  |
| Sensitive | 6 (19.4%) | 15 (31.3%) | 2 (18.2) |  |  |  |
| **Amikacin** |  |  |  |  | *p-*value= **0.05** |  |
| Resistant | 20 (64.5%) | 37 (77.1%) | 8 (72.7%) |  |  |  |
| Intermediate | 2 (6.5%) | 2 (4.2%) | 3 (27.3%) |  |  |  |
| Sensitive | 9 (29%) | 9 (18.8) | 0 (0%) |  |  |  |
| **Levofloxacin** |  |  |  |  | *p-*value= **0.0001** |  |
| Resistant | 25 (80.6%) | 48 (100%) | 8 (72.7%) |  |  |  |
| Intermediate | 5 (16.1%) | 0 (0%) | 0 (0%) |  |  |  |
| Sensitive | 1 (3.2%) | 0 (0%) | 3 (27.3) |  |  |  |

**^1^** Statistically significant *p*-values are bolded and underlined

**Table S3** Distribution of carbapenemases encoding genes of 30 *A.baumannii* isolates with various biofilm formation abilities

| **Genotype** | **Biofilm formation *n* (%row)** | | |  | **Fisher's Exact Test^1^** |  |
| --- | --- | --- | --- | --- | --- | --- |
|  | **Strong** | **Moderate** | **Weak** |  |  |  |
| ***bla*_VIM_** and ***bla*_oxa-23_** |  |  |  |  | *p-*value= **0.0347** |  |
| Both Negative | 3 (42.9%) | 1 (14.3%) | 3 (42.9%) |  |  |  |
| Either Positive | 10 (43.5%) | 12 (52.2%) | 1 (4.3%) |  |  |  |
| ***bla*_NDM_** and ***bla*_oxa-23_** |  |  |  |  | *p-*value= 0.878 |  |
| Both Negative | 4 (36.4%) | 5 (45.5%) | 2 (18.2%) |  |  |  |
| Either Positive | 9 (47.4%) | 8 (42.1%) | 2 (10.5%) |  |  |  |
| ***bla*_VIM_** and ***bla*_NDM_** |  |  |  |  | *p-*value= 0.483 |  |
| Both Negative | 8 (53.3%) | 6 (40%) | 1 (6.7%) |  |  |  |
| Either Positive | 3 (30%) | 4 (40%) | 3 (30%) |  |  |  |
| Both Positive | 2 (40%) | 3 (60%) | 0 (0%) |  |  |  |

**^1^** Statistically significant *p*-values are bolded and underlined

| **A** |  |
| --- | --- |
|  |  |
|  |  |
|  |  |
|  |  |
|  |  |

**Figure S1** Bacterial growth curves of *A.baumannii* isolates in the presence of sub-inhibitory concentrations (½ MICs and ¼ MICs) of **gallic acid,** **(A)** Strong biofilm formers,(**B**) Weak biofilm formers, along with the untreated growth controls

| **B** |  |
| --- | --- |
|  |  |
|  |  |
|  |  |
|  |  |
|  |  |

**Figure S2** Bacterial growth curves of *A.baumannii* isolates in the presence of sub-inhibitory concentrations (½ MICs and ¼ MICs) of **cinnamic acid**, **(A)** Strong biofilm formers,(**B**) Weak biofilm formers, along with the untreated growth controls.

| **A** |  |
| --- | --- |
|  |  |
|  |  |
|  |  |
|  |  |
|  |  |

| **B** |  |
| --- | --- |
|  |  |
|  |  |
|  |  |
|  |  |
|  |  |
